# The confidence-accuracy relationship for lineup decisions holds for the Dutch identification procedure

**DOI:** 10.1371/journal.pone.0284205

**Published:** 2023-04-11

**Authors:** Melanie Sauerland, Nina Tupper, Micol Iannuzzi, Adri G. van Amelsvoort

**Affiliations:** 1 Department of Clinical Psychological Science, Maastricht University, Maastricht, The Netherlands; 2 National Police Netherlands, Working Group Facial Biometrics, Netherlands; Julius-Maximilians-Universität Würzburg, GERMANY

## Abstract

Post-decision confidence from witnesses who make a positive identification decision can serve as a valuable indicator of identification accuracy under certain conditions. International best-practice guidelines therefore recommend asking witnesses to indicate their confidence following a selection from a lineup. Three experiments that used Dutch identification protocols, however, reported no significant post-decision confidence-accuracy association. To examine this conflict between the international and the Dutch literature, we tested the strength of the post-decision confidence-accuracy relationship for lineups that followed Dutch protocol in two ways: we conducted an experiment and re-analyzed two experiments that implemented Dutch lineup protocols. As expected, the post-decision confidence-accuracy relationship was strong for positive identifications and weak for negative identification decisions in our experiment. The re-analysis of the pre-existing data showed a strong effect for positive identification decisions of participants up to the age of 40 years. For exploratory purposes, we also tested the confidence-accuracy relationship between lineup administrators’ perception of witnesses’ confidence and eyewitness identification accuracy. In our experiment, the relationship was strong for choosers and weak for nonchoosers. The re-analysis of pre-existing data showed no correlation between confidence and accuracy, unless we excluded adults over 40 of age. We recommend adapting the Dutch identification guidelines to reflect the current and previous findings on the post-decision confidence-accuracy relationship.

## Introduction

Eyewitness identifications can be valuable in the investigation of crimes, can serve as evidence in court, and can be instrumental in a conviction. Yet, overturned convictions all over the world have shown that it is possible for a sincere witness to identify the police suspect as the perpetrator and be wrong [1–5]. Such identification errors are dangerous because they may lead to the conviction of an innocent suspect, while the true perpetrator may reoffend. Post-decision confidence is one factor that can help assess the accuracy of an identification decision. Yet, current Dutch lineup guidelines advise against collecting confidence statements [6]. Here, we tested the post-decision confidence-accuracy relationship in an experiment that implemented Dutch lineup protocols and re-analyzed two experiments that followed Dutch lineup procedures.

A rich body of research shows that post-decision confidence from witnesses who make a positive identification decision can serve as a valuable indicator of identification accuracy (e.g., [7–10]). For this reason, researchers and best-practice guidelines urge police to ask witnesses to give some rating of confidence immediately following a selection from a lineup [11,12]. This recommendation comes with caveats, however, because for confidence to be indicative of identification accuracy, a few conditions need to be met.

First, the relationship holds only for witnesses who make a selection (i.e., *choosers*), but not for witnesses who reject the lineup (i.e., *nonchoosers*; [8]). Second, the lineup composition needs to be fair because the confidence judgment is inflated if the suspect stands out from the lineup, thus undermining the confidence-accuracy relationship [13]. Third, the confidence judgment should be collected and documented immediately following the identification decision because post-identification feedback from police, other witnesses, and media reports might inflate the confidence judgment [14]. To ensure that the lineup administrator does not provide verbal or non-verbal feedback prior to the confidence judgment, administration of lineups should be double-blind [15]. This means that the lineup administrator has no knowledge of the identity of the suspect and the witness knows that the administrator does not have this knowledge. Finally, the association between confidence and recognition accuracy is weak in children younger than 14 years of age [16,17] and the association might decrease from the age of about 40 years ([18], but see [19]).

According to Dutch identification guidelines [6], lineup administrators should not ask witnesses to provide a post-decision confidence judgment following their identification decision. Specifically, the guidelines from 2013 read: “Witnesses’ own confidence judgments about their [identification] decision say little or nothing. You must therefore not ask about it. The answer is of no use.” (p. 97, [20]). The most recent edition of the handbook provides a somewhat more nuanced statement: “There is […] no guarantee [of accuracy], if a witness indicates that he is confident about his identification decision. Scientific research and our own experience have shown that one’s […] confidence in an identification decision is not always indicative of the accuracy of that decision. After all, a witness who is mistaken can still be very confident […].” (p. 40–41, [6]). Following an elaboration on recent research (e.g., [12]), the handbook concludes that the confidence-accuracy “is too nuanced to provide an absolute judgment on the accuracy of a recognition decision in an individual case.” Indeed, Dutch legal psychologists have long advised against the use of confidence judgments for assessing identifications, based on the idea that the association between confidence and accuracy was weak [21,22]. This assessment was supported by three experiments that used Dutch lineup protocols and found a weak relationship between confidence and identification accuracy [23–25].

The discrepancy between Dutch studies that find a weak confidence-accuracy relationship and the vast literature that finds a strong relationship could stem from differences in methodology, analysis, or both. Specifically, this relationship can be altered by procedural differences in lineup construction or administration or misinterpreted based on choices in statistical analyses. To explore the apparently weak confidence-accuracy relationship in Dutch experiments, we review both the methodology of Dutch identification procedures and the statistical analyses used to interpret the results.

### Lineup construction and administration in the Netherlands

The Dutch Handbook for identification procedures ([6]; Handbook henceforth) details protocols for the construction and administration of lineups in the Netherlands since 1994. For example, according to the Handbook, lineups can include between five and eleven fillers, witnesses may view the lineup simultaneously or sequentially, and lineups can be administered live, or using photos or videos. In practice, sequential photo lineups come into use most frequently, whereas live lineups are rare.

All lineup variations follow several important protocols. First, before administration, police test the lineup to ensure its fairness. At least two mock-witnesses similar in age, origin and background, and gender to the witness receive the witness’ perpetrator description and view the lineup. If there are several witnesses who differ on these characteristics, more mock-witnesses may participate. It is their task to point out lineup members who stand out to them (if any) and to explain why. If a mock-witness selects the suspect, fillers are replaced to improve the similarity between the suspect and the fillers. Selected fillers are removed or replaced, as long as the resulting final lineup size is still six or larger. This procedure deviates from the mock-witness paradigm that researchers often use to test the fairness of a lineup [26]. In this procedure, approximately 20 to 30 non-witnesses receive a person description of the perpetrator and the lineup and point out the lineup member who best matches the description. If the lineup is fair, each lineup member should be selected approximately equally often. Differences between these two procedures mostly go back to logistic reasons.

A second important feature of Dutch lineups is that they are double-blind [27]. For this purpose, a lineup supervisor constructs the lineup and prepares the witness for the procedure. Subsequently, the lineup administrator–who does not know which lineup member is the suspect–presents the lineup to the witness. When there are several witnesses, different lineup administrators may be involved. Furthermore, a computer program determines a random order of lineup members for each witness. Additionally, witnesses receive written instructions that inform them that the lineup administrator is unaware of the identity of the suspect.

#### The confidence-accuracy relationship in Dutch Lineups

Three experiments that examined the confidence-accuracy relationship with the protocols and materials specific to the Netherlands did not report a significant association between post-decision confidence and identification accuracy. In all of the three experiments, participants witnessed a staged event and then viewed an 8 or 9-person target-present or target-absent lineup [23–25]. The lineups were constructed and administered in accordance with Dutch protocols (i.e., [6] and earlier editions). In line with international recommendations for lineup administration [11,28], instructions included the caution that the perpetrator may or may not be present the lineup, that, if unsure, no selection should be made, and that, if the witness happened to know anyone in the lineup, they should point that out. Lineups were administered double-blind. Next to target presence, these experiments manipulated lineup mode (live vs. photo vs. video; sequential vs. simultaneous; [24]), context reinstatement (no context vs. context reinstatement; [23]), or tested the effect of age on identification performance (25–50 vs. 65 years and older; [25]). Following their lineup decision, participants indicated their post-decision confidence on a 7- or 5-point scale.

In the first experiment (*N* = 337), post-decision confidence was not a statistically significant predictor of identification accuracy when entered with age, gender, level of education, and lineup administrator confidence in three separate regression models for live, photo, and video lineups [24]. In the second experiment (*N* = 104), a 2 (identification accuracy: accurate vs. inaccurate) x 5 (post-decision confidence level: 1 through 5) chi-square test did not return a significant effect [23]. In the most recent experiment (*N* = 109), post-decision confidence ratings between 1 and 5 were classified as *low* and post-decision confidence ratings between 6 and 7 as *high*. A 2 (accuracy: accurate vs. inaccurate) x 2 (confidence level: high vs. low) chi-square test returned no significant effect [25].

The lack of a statistically significant confidence-accuracy relationship across those three experiments is in contrast to a large literature that documents a strong confidence-accuracy relationship for adults’ positive lineup decisions [8,9,12,29]. One possible explanation for the Dutch findings could be that specific protocols applied in the Netherlands undermine the confidence-accuracy relationship. Indeed, witnesses in the Netherlands receive a leaflet with written instructions prior to the presentation of the lineup that warns them that it is difficult to recognize a person from a photograph, that, if in doubt, they should not identify anyone, and that it is possible that the person who committed the theft is not present in the lineup. The first two elements of these instructions are unique to the Netherlands. It is possible that these instructions, combined with the caution that the perpetrator may not be present discourage low confidence identifications to such an extent that post-decision confidence judgments do not have added value.

However, Dutch lineup construction and administration protocols are largely in line with international recommendations [11,28] in that they are guided by the idea that the suspect or foils should not stand out and that the lineup should not be suggestive. Experiments in the international literature typically implement these recommendations across experimental conditions and such experiments have consistently found a strong confidence-accuracy relationship for positive identification decisions with these recommended protocols. Therefore, under the pristine testing conditions of Dutch protocols, a strong post-decision confidence relationship for positive identification decisions would be expected.

Another explanation for the observed divergence between the international and the Dutch findings on the confidence-accuracy relationship concerns three statistical issues in some or all of the Dutch experiments (cf. [30]). Most critically, none of the three experiments reported separate analyses for positive and negative identification decisions. This differentiation is crucial because the confidence-accuracy relationship is strong for choosers but weak for nonchoosers [8,12]. Furthermore, the sample sizes of two experiments were likely too small to detect an effect [23,24]. For example, in Eigenhuis and van Amelsvoort [23], there were 52 choosers, split across the two context reinstatement conditions. For a 2 x 5 chi-square test, 133 choosers (per context condition) would have been necessary to be able to detect a medium effect with a power of .80, and 48 choosers to detect a large effect [31]. The final experiment suffered from a loss of information by turning confidence into a dichotomous variable [25]. Doing so obscures the nuance of observing accuracy at various levels of confidence, which is the focus of the most recent statistical developments in the field.

### Lineup administrator confidence in identification decisions

In addition to witnesses’ confidence judgements, the Dutch experiments also collected confidence judgments from lineup administrators. Such judgements served as a test of blind observers’ capacity to assess identification accuracy. The results were mixed. In two of the experiments, decisions judged “reliable” (confidence ratings 6 or 7), were more often accurate than “less reliable” decisions (confidence rating 1–5; [24,25]). The authors concluded that lineup administrators’ judgments of witnesses’ confidence were better predictors of identification accuracy than witnesses’ confidence judgements. Kerstholt and van Amelsvoort [24] additionally suggested that providing lineup administrators with feedback might improve the relationship further. However, the third Dutch experiments reported no significant relationship between lineup administrator’ ratings of eyewitness confidence and identification accuracy [23]. The shortcomings of these three experiments mentioned above (small sample sizes, treating confidence as a dichotomous variable, no differentiation between choosers and nonchoosers) also apply to these analyses.

A recent North American experiment examined how the ability of witnesses and lineup administrators to assess identification accuracy varied as a function of witness justifications [32]. Witnesses provided one of five different types of justifications: familiarity (e.g., “He looks familiar”), observable feature/s (e.g., “I remember his eyes”), unobservable (e.g., “He looks like someone I know from school”), and recognition (e.g., “I remember seeing him”). The predictive value of *perceived* eyewitness accuracy differed as a function of the observer. Eyewitnesses’ confidence ratings were good predictors of their own accuracy—when their identification decision was accompanied by any justification except for familiarity. Familiarity justifications were associated with overconfidence errors. However, lineup administrators correctly predicted witness accuracy only when witnesses provided an unobservable justification for their choice. Lineup administrators interpreted other types of justifications more variably, therefore leading to inconsistent results. This finding suggests that justifications that accompany witnesses’ identification decisions can influence the diagnosticity of witness and lineup administrator confidence ratings.

One possible explanation for the divergent findings of the Dutch experiments on the predictive value of lineup administrator confidence includes the involvement of very experienced lineup administrators [24] vs. less experienced administrators [23]. Moreover, interindividual differences in the non-verbal behavior of witnesses and differences in the interpretation of these behaviors by the administrators might also play a role [23,32]. In Dutch practice, investigators are likely to discard identifications that they perceive as less reliable, whereas they might place considerable value on identifications that they perceive to be reliable [24]. Therefore, a lineup administrator’s confidence judgment could potentially contribute to shifts in investigations away from guilty culprits and towards wrongful prosecutions [33]. To add to the currently small database on the relationship between lineup administrators’ confidence ratings and eyewitness identification accuracy, we also collected lineup administrators’ post-decision confidence ratings. Additionally, we reanalyzed the data of the two Dutch Experiments for which data were available [23,25].

### Research questions and hypotheses

We tested the confidence-accuracy relationship in lineups that followed Dutch protocol in two ways: we conducted an experiment that followed the Dutch lineup procedure and we re-analyzed data from two earlier experiments that used Dutch identification protocols ([23,25]; unfortunately, the data from Kerstholt and van Amelsvoort [24] were no longer available). In our experiment, participants viewed a stimulus film showing a man or a woman who stole the wallet of another person at a bus stop. After a short retention interval, participants viewed a target-absent or target-present lineup of the thief. We expected to find a strong post-decision confidence-accuracy relationship for positive identification decisions both in our own experiment and in the re-analysis, with the exception of the older participants [18]. We expected no such effect for lineup rejections. For exploratory purposes, we also tested the confidence-accuracy relationship for lineup administrators. To this end, lineup administrators in our experiment provided confidence ratings following witnesses’ identification decisions and we reanalyzed the two Dutch experiments for which data were still available [23,25].

## Methods of current experiment

The data of our experiment are available at https://doi.org/10.34894/XYUBY8.

### Participants

We started data collection in the fall of 2019. Due to the COVID-19 pandemic, data collection was halted in the spring of 2020. We then collected as many participants as possible by the end of the academic year 2020/21. Three-hundred-and-eleven participants (61 male, 249 women, 1 diverse) took part in the experiment. They learned about the experiment through the University’s participant recruitment platform (SONA), online advertisements, flyers at various university faculties, and during open days at the university in which prospective students tour the faculty. The experiment was advertised as a study where participants would find out what it was like to be a police witness. The advertisement did not mention eyewitness identification specifically.

We excluded 12 participants because they differed in origin from the targets in the stimulus films in order to prevent other-group bias as much as possible [34]. We further excluded three participants because they previously knew a lineup member, two participants because their confidence was not recorded by error, and one participant because their phone rang during the administration of the lineup. Thus, the final sample consisted of *N* = 293 participants, (59 men, 233 women, 1 diverse; *M*_*age*_ = 22.11 years; 16–61 years [age of consent is 16 years in the Netherlands]; *SD* = 7.83; *Mdn* = 20). Participants were students (75.8%), had completed higher vocational education (11.9%), university education (7.5%), medium vocational education (3.1), or lower vocational education (1.7%). Participants received either 0.5 participation credits or a 5 € gift voucher. Data of 259 participants could be used for the association between lineup administrators’ confidence and witnesses’ identification accuracy. The smaller sample size for these analyses is due to the following: when testing during the open days, we had to involve two lineup administrators who were familiar with the hypothesis on lineup administrators’ confidence judgments. Therefore, we did not include those administrators’ confidence data. This experiment was approved by the standing ethical committee of the Faculty Psychology and Neuroscience at Maastricht University (code OZL_231_140_12_2020).

### Design

The experiment used a one factorial design with two levels: target presence (target-present vs. target-absent). The position of the target in the lineup varied between positions 2, 5, and 6 (female thief) and positions 3, 6, and 8 (male thief). We counterbalanced the roles of the two actors (female thief vs. male thief; female victim vs. male victim).

### Materials

#### Stimulus films

Participants viewed one of two stimulus films that depicted a nonviolent mock-theft of a wallet. The script of the two films was identical, with the two actors switching roles (thief vs. victim) between versions. The action can be described as follows: person 1 (who will become the thief) is standing at a bus stop when person 2 approaches the bus stop on crutches. Person 2 takes a seat, takes something out of their backpack and leaves the backpack behind when they walk to the bus stop information board. At this time, person 1 opens the backpack, takes out a wallet, unnoticed by person 1, and walks away. The films were 1:24 and 1:37 min long and the perpetrator’s face was visible in close-up for approximately 19 sec.

#### Lineup construction

The target-absent and target-present lineups consisted of one suspect (innocent or guilty) and seven fillers. Lineup photographs showed each lineup member from the shoulders up in front of a white background; including one portrait picture and a 45° angle profile picture for each lineup member. The two pictures of each lineup member were presented simultaneously; the different lineup members were presented sequentially.

We constructed the lineups in line with Dutch police protocol [6]. For each target, we created a person description that included information about age, body shape, hair color, and hair length. Following Dutch police procedure, we presented each target and a selection of possible fillers to four mock-witnesses (two men, two women) who were similar in age and origin to our target population (i.e., students). They were informed that a theft had occurred and received a general perpetrator description prepared by women (for female test observers) or men (for male test observers), respectively (e.g., She’s about 20 years old, has long, brown hair and a slim figure). It was their task to point out anyone who stood out (if anyone), for whatever reason. In the first round, the test observers pointed out several fillers who were subsequently dropped or replaced. The final selection of eight fillers (one serving as replacement for target-absent lineups) was established following a second round with test observers.

We established the *effective lineup size* by means of the mock-witness paradigm [26]. Independent samples of mock-witnesses (*n*s between 30 and 38) who had not seen the stimulus event, read a target description similar to the descriptions used for the Dutch police protocol described above and viewed the lineup. Mock-witnesses then selected the person from the lineup who matched the description best. Tredoux’s *E* (i.e., the effective lineup size) ranged from *E* = 5.8 to 7.3 (of a possible 8), thereby marking them as a fair selection [35,36].

#### Lineup administration

We administered the lineups in line with Dutch police protocol [6]. Prior to the lineup presentation, participants received the standard Dutch police lineup information leaflet [6] that informed them that they a) would see a sequential lineup, b) would see the selection once, and c) should immediately say so if they saw the person who committed the theft. The leaflet emphasized that 1) the lineup administrator did not know the lineup members nor who was suspected of the theft and that the fillers were innocent citizens; 2) it was difficult to recognize a person from a photograph; 3) if in doubt, they should not identify anyone; and 4) it was possible that the person who committed the theft was not present in the selection.

The lineup was presented sequentially, using PowerPoint. Following Dutch procedure, each lineup member appeared on screen for 4 seconds followed by 3 seconds of a black screen. Lineup members were numbered 1–8. Lineups could only be viewed once and could be paused on the black screen. Participants first viewed two test photographs with the same timing as the actual lineup photographs. As a reminder, participants were then again warned that it was possible that the person wanted was not part of the selection and that they should not point out anyone if they were in doubt. These instructions were simultaneously given both orally and in writing, as part of the PowerPoint [37].

If participants made a selection, the lineup administrator wrote down the number of the photograph. In line with Dutch procedure, the lineup then continued until the last lineup member. If the witness selected someone, lineup administrators covertly recorded their confidence in the participant’s lineup decision on a scale from 0 to 100% and then asked the participant how confident they were about the decision they had just made. If the participant did not select someone, administrators documented their confidence in the lineup rejection at the end of the lineup, just before asking participants for their confidence.

#### Blinding administrators

The blinding of administrators was accomplished in two ways. The lineup was presented on two screens–one for the participant and one for the lineup administrator. The administrator’s screen was covered during lineup presentation such that only the number of the presented lineup member was visible (1–8). Additionally, and to avoid lineup administrators learning the likely position of the target over time, suspect positions varied between three different positions for each target and across film versions. Lineups were created by an experiment supervisor who was the only person aware of the suspect positions and counterbalancing scheme and who provided the lineup as a blinded file.

### Procedure

All participants were tested individually and the identification procedure was video recorded. After signing the written informed consent form, participants viewed the stimulus film. During the 10-minute retention interval, participants provided demographic information and completed several unrelated filler tasks on the computer. Then, the lineup was administered. Afterwards, participants were asked what they thought the purpose of the experiment was prior to participating. In case the participant mentioned identification, they were asked to indicate the reason for their assumption. Participants furthermore indicated whether they knew any of the lineup members. A ‘Yes’ answer led to the exclusion of their data. Before leaving, participants were asked not to talk about the contents of the experiment to avoid bias in future participants. Participants then received a full debriefing and reimbursement.

## Results

### Current experiment

#### Overview of lineup outcomes

Seventy-one participants selected a lineup member from the lineup, 222 participants rejected the lineup. The correct identification rate for target-present lineups was 55/142 (i.e., 38.7%). The false selection rate in target-absent lineups was 13/151 (i.e., 8.6%). Considering that only one of the eight lineup members would be the innocent suspect, the false identification rate was 8.6/8 = .011%. The three remaining selections were foil selections in target-present lineups, and hence known errors. Descriptive statistics of confidence can be found in Table 1.

**Table 1:** Descriptives of choosers’ and nonchoosers’ identification accuracy and witnesses’ and lineup administrators’ post-decision confidence.

	*M*	*SD*	95% CI
Identification accuracy			
Choosers	77.5		67.5–87.4
Nonchoosers	62.1		55.7–68.6
Witnesses’ post-decision confidence			
Choosers	79.5	14.2	76.1–82.8
Nonchoosers	69.2	20.0	66.6–71.8
Administrators’ post-decision confidence			
Choosers	70.7	23.9	64.5–76.8
Nonchoosers	74.3	20.4	71.4–77.1

CI = Confidence interval.

*Witnesses’ confidence ratings*: *Correlations between post-decision confidence and identification accuracy*. For choosers (*n* = 71), the smallest effect size we had 80% power to detect was *r* = .28 for a one-tailed test **[31]**. For nonchoosers (*n* = 222), the smallest effect size we had 80% power to detect was *r* = .17. The confidence-accuracy correlation for choosers was *r*(69) = .41, *p* < .001, indicating a large effect. For nonchoosers, the effect size was small, *r*(220) = .19, *p* = .004.

**Fig 1:**
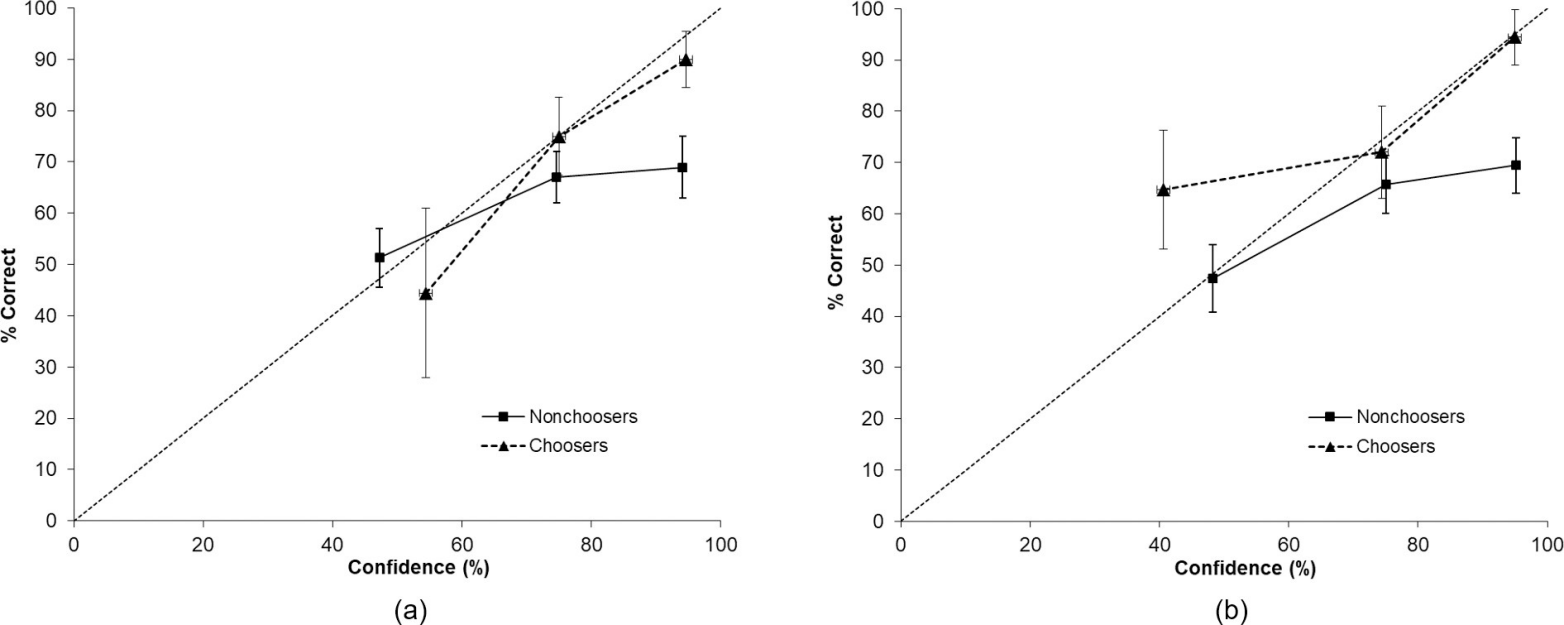
Eyewitnesses’ (a) and Lineup Administrators’ (b) Post-Decision Confidence-Identification Accuracy Calibration Curves split for Choosers and Nonchoosers (and Standard Error).

*Witnesses’ confidence ratings*: *calibration analyses and calibration curves for witness confidence*. Fig 1(A) displays witnesses’ confidence-accuracy calibration curves for choosers and nonchoosers. For these analyses, we rounded confidence ratings to fit an 11-point Likert scale ranging from 0 to 100% (i.e., 0%, 10%, 20%, …, 90%, 100%). In line with earlier work, we collapsed confidence categories between 0 and 60% (choosers: *n* = 9; nonchoosers: *n* = 76), 70 and 80% (choosers: *n* = 32; nonchoosers: *n* = 88), and 90 and 100% (choosers: *n* = 30; nonchoosers: *n* = 58) to account for the small number of participants per confidence category [38–40].

Choosers’ calibration curve followed the diagonal neatly, with slight overconfidence in the high confidence category, perfect calibration in the mid category, and some overconfidence in the lower part of the scale. Choosers’ overall calibration was very high at .0022. Over-/underconfidence across confidence categories was .032, indicating a general tendency for some overconfidence. The resolution index *NRI* = .119 signified a moderate capacity for differentiating between accurate and inaccurate chooser decisions.

For nonchoosers, the curve was much flatter than the diagonal, displaying strong overconfidence especially in the high confidence category. Nonchoosers’ overall calibration was *C* = .0194 and the overall *O/U* = .082. The resolution index *NRI* = .026 indicated a weak capacity to differentiate between accurate and inaccurate nonchooser decisions. The upper part of Table 2 reports the statistical calibration indices *C*, *O/U*, and *NRI* with jackknife *SE*s (following [41]) and 95% CIs.

**Table 2:** Calibration statistics for choosers and nonchoosers for eyewitness and lineup administrator confidence ratings of eyewitness accuracy.

Eyewitness ratings						
	Choosers			Nonchoosers		
		Jackknife *SE*	95% CI		Jackknife *SE*	95% CI
*C*	.0022	.004	.000; .011	.0194	.035	.000; .088
*O/U*	.032	.046	-.059; .123	.082	.033	.018; .146
*NRI*	.119	.090	.000; .296	.026	.022	.000; .070
Lineup administrator ratings						
*C*	.0167	.016	-.015; .049	.0269	.025	-.022; .076
*O/U*	-.057	.054	-.163; .050	.128	.035	.060; .197
*NRI*	.081	.057	-.031; .192	.037	.028	-.018; .091

*Posterior-by-prior Bayesian curves*: *Considering Base Rates of Target-Presence*. In the current experiment, half of the lineups presented an innocent suspect instead of the true perpetrator. This means that the base rate, or prior probability that the suspect was the perpetrator, was 50%. In the final stage of the analyses, we addressed the possible concern that the confidence-accuracy relationship may hold only for certain base rates that the suspect is the perpetrator. To this end, we created posterior-by-prior Bayesian curves that map the probability that a suspect identification was accurate (i.e., that the suspect was the perpetrator) across all possible base rate values from 0% (all lineups displayed an innocent suspect) to 100% (all lineups displayed a guilty suspect; cf. [12,42–44]). We created one curve for highly confident choosers (90–100%) and one for less confident choosers (0–80%) The identity line shows where the data would fall if an identification was non-diagnostic.

**Fig 2:**
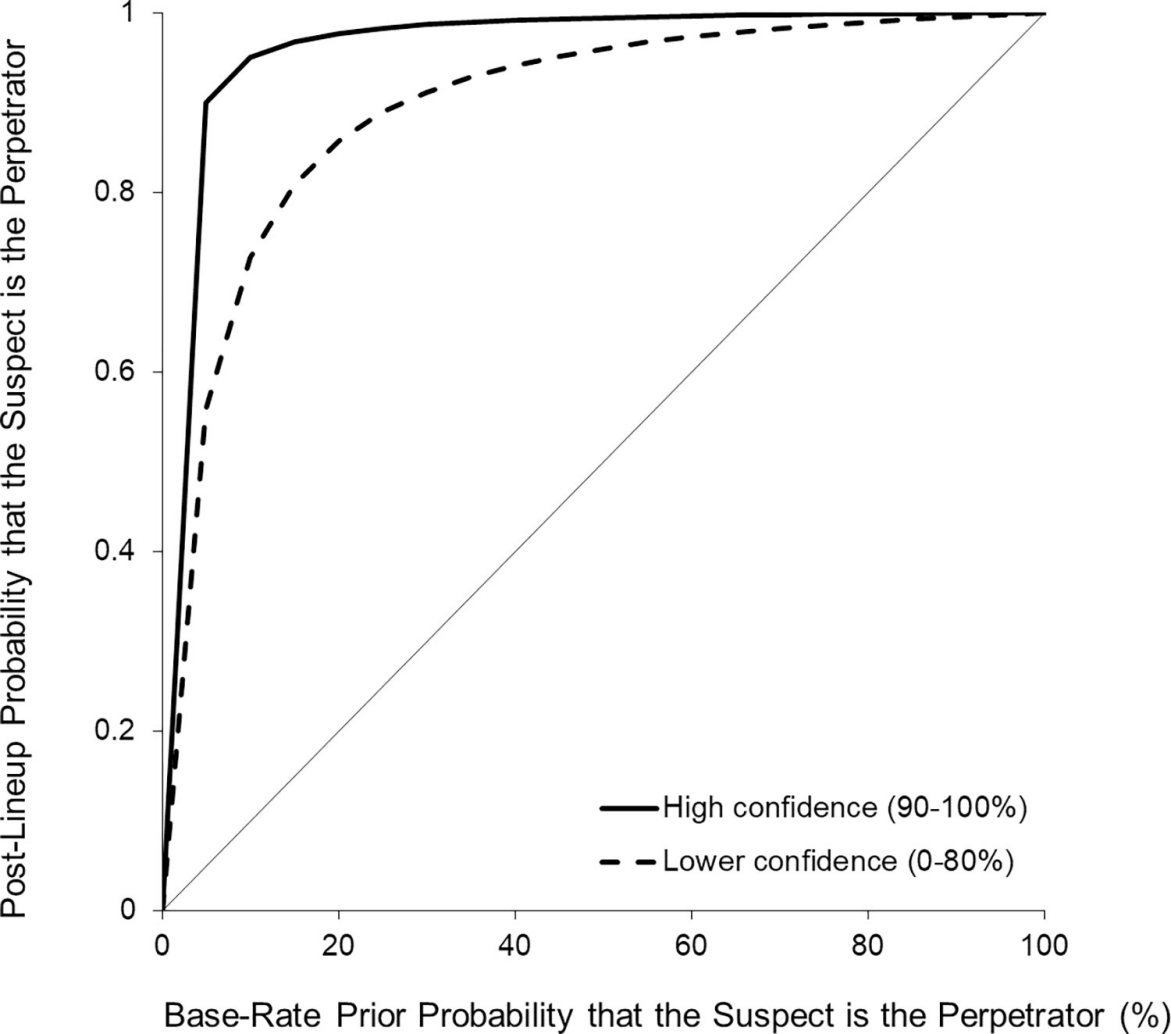
Post-lineup probability that the suspect is the perpetrator as a function of the base rate of target-presence for highly confident and less confident participants.

Three important observations can be made from Fig 2. First, both curves are well above the identity line, indicating that identifications were diagnostic of guilt. More importantly, as expected, the height of the curve for confident decisions was far above the curve for less confident decisions. Notably, the high-confidence curve comes remarkably close the upper left corner. Third, the probability that the identified suspect was the perpetrator for highly confident decisions remained high (above 90%) until the base rate dropped below 5%. For less confident decisions, accuracy dropped below 90% at a base rate of 25%. Or in other words, whereas highly confident decisions were still highly accurate (90%) at a base rate of a mere 5%, less confident decisions were much less accurate (55.8%) at this base rate.

*Lineup Administrators’ Confidence Ratings*: *Correlations*. Lineup administrators’ post-identification confidence correlated positively with witnesses’ identification accuracy for choosers, *r*(58) = .30, *p* = .020, indicating a moderate effect size. For nonchoosers, the effect was weak, *r*(197) = .18, *p* = .011.

We also wanted to study whether informing lineup administrators about non-verbal accuracy cues could be used as a potential tool for enhancing their ability to discriminate between accurate and inaccurate eyewitnesses. Midway through data collection, lineup administrators underwent a 15-min briefing. During this briefing, they learnt about the decision time-accuracy relationship [9,45], absolute vs. relative judgments [46], and automatic vs. effortful identification processes [47,48]. Correlation analyses split by briefing (pre-briefing vs. post- briefing) yielded the following: choosers: *r*(21) = .25, *p* = .246, *r*(30) = .32, *p* = .075; nonchoosers: *r*(86) = .15, *p* = .16, *r*(94) = .19, *p* = .063, respectively. The sample is *N* = 239 here, because we only included data of experimenters who collected data prior to and following the training.

*Lineup Administrators’ Confidence Ratings*: *Calibration Analyses*. Fig 1(B) displays administrators’ confidence-accuracy calibration curves for choosers and nonchoosers. The confidence categories were, once again, collapsed between 0 and 60% (choosers: *n* = 17; nonchoosers: *n* = 57), 70 and 80% (choosers: n = 25; nonchoosers: *n* = 70), and 90 and 100% (choosers: *n* = 18; nonchoosers: *n* = 72).

Lineup administrators’ calibration curve for choosers looked quite similar to the witnesses’ curve in the high confidence and mid categories, with almost perfect calibration in the high confidence category and slight overconfidence in the mid category. In the low confidence category, lineup administrators showed strong underconfidence. Calibration for choosers’ decisions overall was good at *C* = .0167. Over-/underconfidence across confidence categories indicated a general tendency for some underconfidence of lineup administrators, *O/U* = -.057. The resolution index *NRI* = .081 was indicative of moderate capacity of discrimination between accurate and inaccurate chooser decisions.

Similar to witnesses’ confidence judgements, the curve was much flatter for nonchoosers, displaying strong overconfidence in the high confidence category, some overconfidence in the mid category, and perfect calibration in the low confidence category. Nonchoosers’ overall calibration was *C* = .0269 and the overall *O/U* = .128. The resolution index was *NRI* = .037, indicating a weak capacity to differentiate between accurate and inaccurate nonchooser decisions. The lower part of Table 2 reports the statistical calibration indices *C*, *O/U*, *NRI* with jackknife *SE*s, and 95% CIs.

### Re-analyses of previous experiments

For both experiments [23,25], we computed the post-decision confidence-accuracy correlations for positive identification decisions and for rejections. We also conducted separate analyses for older vs. younger participants because the confidence-accuracy relationship can be stronger for younger adults compared with older adults (e.g., [18]). Additionally, we computed the post-decision confidence-accuracy correlations for confidence ratings expressed by the lineup administrators. The sample sizes, however, did not allow for calibration or confidence-accuracy-characteristic analyses, or for a statistical comparison of the different correlation coefficients. Table 3 shows an overview of the confidence-accuracy correlations for choosers and nonchoosers for both experiments.

**Table 3:** Witnesses point-biserial confidence-accuracy correlations with p-values and sample sizes computed split across choosers and nonchoosers for two experiments.

	Eigenhuis & van Amelsvoort (2015)			Thijssen & van Amelsvoort (2016)		
	*r*	*p*	*n*	*r*	*p*	*n*
Choosers						
overall	.04	.779	52	.13	.389	44
up to age 30	**.36**	.128	19	.*27*	.390	12
up to age 40	**.36**	.114	21	**.37**	.148	17
41 and older	-.18	.348	31	.02	.924	27
51 and older	.08	.772	15			
61 and older				.05	.796	26
71 and older				.*25*	.429	12
Across age with context reinstatement	.*26*	.165	30			
Across age no context reinstatement	*-*.*26*	.238	22			
Nonchoosers						
overall	.07	.608	53	.13	.426	41
up to age 30	.01	.969	18	<.01	>.999	16
up to age 40	.09	.692	23	-.01	.979	26
41 and older	.03	.882	30	.*34*	.212	15
51 and older	.07	.780	18			
61 and older				.**40**^a^	.175	13
with context reinstatement	< .01	>.999	25			
no context reinstatement	.14	.481	28			

Correlations in italics mark moderate effect sizes; correlations in bold mark large effect sizes.

^a^ we do not report *r* for 70 years and older, because of the small sample size (*n* = 6). The grey cells indicate that there are no data for this age group.

#### Eigenhuis and van Amelsvoort (2015) [23]

Participants (*N* = 105, 61 women, age range = 17–67, *M*_age_ = 40.7, *Mdn*_age_ = 45) were assigned to a context reinstatement (*n* = 55) or no context reinstatement condition (*n* = 50). Overall, the confidence-accuracy correlation was weak for both choosers and nonchoosers (see Table 3). To test whether age played a role in the confidence-accuracy correlation, we computed separate correlations for participants up to the age of 30 and 40 and for participants older than 40 and 50 years (cf. [18]). Those analyses revealed that the confidence-accuracy correlation was strong for choosers up to the age of 40, but weak for choosers who were older than 40 years. There was not enough power to compare the correlation coefficients on an inferential level. For choosers in the context reinstatement condition, there was a moderate, positive association between post-decision confidence and accuracy, but for choosers without context reinstatement, there was a moderate negative association. As expected, nonchoosers’ confidence-accuracy correlation was weak, regardless of condition and participant age.

Lineup administrators’ confidence ratings and participants’ identification accuracy were not or only weakly correlated for both choosers, *r*(50) = -.06, *p* = .667, and nonchoosers, *r*(51) = -.03, *p* = .819, respectively.

#### Thijssen and van Amelsvoort (2016) [25]

Participants were assigned to two groups, based on their age (25 to 50 vs. 65 to 86 years; *N* = 105, 61 women, age range = 17–86, *M*_age_ = 40.7, *Mdn*_age_ = 45). The findings were quite similar to Eigenhuis and van Amelsvoort [23]. Again, the confidence-accuracy correlation, not considering age, was weak for both choosers and nonchoosers (see Table 3). We then computed separate correlations for participants up to the age of 30 and 40 and for participants older than 40, 60, and 70 years. Those analyses revealed a moderate effect size for choosers up to the age of 30 and a large effect size for choosers up to the age of 40. The confidence-accuracy correlation was weak when participants were older than 40 or 60 year old. For choosers older than 70, the effect size was moderate. The sample size of this group was very small though (*n* = 12). There was not enough power to compare the correlation coefficients on an inferential level. Again and as expected, nonchoosers’ confidence-accuracy correlation was weak, regardless of participant age, with the exception of nonchoosers older than 60 years. The sample size of this group was again very small (*n* = 13).

The correlation between lineup administrators’ confidence ratings and participants’ identification was strong for choosers, *r*(42) = .36, *p* = .017, indicating a large effect size. The correlation coefficient was very small for nonchoosers, *r*(38) = -.08, *p* = 618.

## Discussion

We tested the strength of the post-decision confidence-accuracy relationship for lineups that followed Dutch protocol in two ways: we conducted a new experiment and re-analyzed two experiments that used Dutch lineup protocols. As expected, the post-decision confidence-accuracy relationship for positive identifications in our experiment was strong. The re-analysis [23,25] showed a strong effect for positive identification decisions of participants up to the age of 40 years. These findings are in line with a vast international literature on the post-decision confidence-accuracy relationship and recent insights on the possible effects of age on the post-decision confidence-accuracy relationship.

The post-decision confidence-accuracy relationship was strong for positive identification decisions in this experiment that followed Dutch identification protocols [6]. Different methods of approaching the data (correlation, calibration, confidence-accuracy-characteristic analyses, posterior-by-prior Bayesian curves) all led to this conclusion. The post-decision confidence-accuracy relationship was weak for negative identification decisions. These findings replicate a common finding in eyewitness identification research [7,8,10,29]. Importantly, our findings suggest that the extensive cautioning instructions used in the Netherlands might reduce but do not entirely prevent low confidence identifications. These low confidence identifications were less likely to be accurate compared with medium and high confidence identifications. Thus, our findings confirm the beneficial value of eliciting post-decision confidence judgments for positive identification decisions even when implementing methodology that reflects Dutch lineup construction and administration protocols.

The results of the re-analyses of two experiments that used Dutch lineup procedure were in line with the findings of our experiment, but the overall result pattern was complex. Specifically, age affected the post-decision confidence-accuracy correlation for positive decisions in both experiments [23,25]. The effect size was strong when participants were 40 years or younger, but for participants between 41 and 60 years, the post-decision confidence-accuracy relationship vanished. For participants between 71 and 86 years, the effect size was moderate [25]. However, the number of positive identification decisions in this group was quite small. Although this pattern of results closely matches the findings of a field experiment conducted in Germany that reported both correlation and calibration analyses for participants aged 15 to 83 years [18], these findings should be viewed with caution, because of the very small sample sizes per age group. Indeed, another experiment that included 2,670 participants between 18 and 95 years of age did not report negative effects of age on the confidence-accuracy relationship for fair lineups [19]. More research is needed to be able to draw conclusive inferences about the effect of age on the confidence-accuracy relationship.

For exploratory purposes and to test the usefulness of a common Dutch procedure, we also analyzed the confidence-accuracy relationship between lineup administrators’ confidence and eyewitnesses’ decision accuracy. We tested this in the new experiment and re-analyzed existing data of two experiments that used Dutch lineup protocols. In our experiment, this relationship was strong for positive identifications and weak for negative identifications. In fact, the calibration and CAC curves for witnesses and lineup administrators looked strikingly similar, with the exception of the lowest confidence category. The re-analysis of the two experiments that used Dutch lineup procedure supported what the authors had originally reported, that is, a large effect size for Thijssen and van Amelsvoort [25] and a weak effect size in Eigenhuis and van Amelsvoort [23]. Our findings are in line with two previous experiments that reported good predictive value of lineup administrators’ confidence ratings [24,25], but contrary to one other experiment [23]. If replicated in future studies, lineup administrators’ confidence ratings could be useful, for example in the absence of witness confidence ratings. Double blind lineup administration is likely one important premise of this relationship. Future research should further investigate the usefulness of lineup administrator confidence judgments for predicting eyewitness identification accuracy and possible limiting conditions of such a relationship.

### Limitations

This work has several limitations. Due to the COVID-19 pandemic, the collected sample of choosers was smaller than initially intended. This could have an effect on the stability of the calibration curve if we collapsed the data across five confidence categories as we initially planned. To address this issue, we followed previous research [38,40] and collapsed the data across three confidence categories to preserve the stability of the calibration curve with the available sample size. The number of participants in the lowest confidence category (0–60%) was still very small (*n* = 9). While impractical for statistical analyses, a low number of (very) low post-decision confidence selections is highly desirable for preventing innocent suspect identifications and might be a result of the elaborate warning instructions witnesses receive in the Netherlands. Importantly, there was still a wide variety in participants’ post-decision confidence judgments and a sufficient number of participants who displayed moderate and high post-decision confidence.

Overall, choosers’ identification accuracy in the current experiment was quite high at 77.5%. For a comparison, the average accuracy for lineup performance as reported in meta-analyses revolves around 50% [49–51]. Additionally, choosers often (42%) fell into the highest confidence bin of 90–100% confidence. Combined, these findings could suggest that the current sample performed quite high, compared to other samples. Our sample consisted mostly of university students (78%), but a significant minority were non-students with a much broader age range (16 to 61 years) than most eyewitness studies. With fewer students and more older adults in the sample, one would expect weaker, not stronger performance, compared to other studies. Thus, the attributes of sample cannot explain the high performance observed in this experiment.

Some specifics of the Dutch procedure could explain the relatively strong performance observed. In line with Dutch guidelines, participants received two warnings that they should only make a selection if they were sure enough, one prior to and one during the lineup. The warning during the lineup included written and oral instructions. The inclusion of the oral instructions in the Dutch procedure is based on one experiment that showed that oral instructions reduced false selections from target-absent lineups under certain conditions [37]. The beneficial value of oral instructions in addition to written instructions has not been researched further since the original study. It is conceivable, though, that the repeated written warnings in combination with the oral warning strengthened the effect of the cautioning instruction, resulting in fewer wrongful selections and more high-confidence selections. Our results point into this direction. A direct comparison between different formats of cautioning is necessary, however, before we can draw firm conclusions about the specific effects of the Dutch variant of those instructions.

The re-analysis of the existing Dutch experiments was limited by the small sample sizes and the confounding of context reinstatement and age in one of the experiments [23]. As a result, our re-analysis was confined to computing correlation coefficients, but power was insufficient for comparing these coefficients with each other or for more advanced methods such as calibration of confidence-accuracy-characteristic analyses. These limitations, in combination with the confounding factors age and context reinstatement make it difficult to draw conclusions about the confidence-accuracy relationship from one of the experiments [23]. The re-analysis of the second experiment [25], indicated a strong, positive confidence-accuracy relationship for positive identification decisions.

### Recommendations for policy and practice

Our results provide evidence that post-decision confidence can serve as a meaningful indicator of the accuracy of positive identification decisions for lineups following Dutch protocol. We recommend adapting the relevant Dutch guidelines on police procedures to reflect these findings. Specifically, we recommend that the guidelines implement instructions to collect immediate post-decision confidence judgments from adult witnesses who make a positive identification decision. Post-decision confidence provided by children or for negative identification decisions is not a meaningful indicator of the accuracy of the decision. Adult witnesses should provide a confidence judgment immediately following the identification decision and without any feedback provided to them. It goes without saying that, in the future, the Dutch (and any other) guidelines should take into consideration new insights about the confidence-accuracy relationship, for example about the impact of age and estimator variables [18,19,52,53].

To place the post-decision confidence rating into context for investigators, lawyers, prosecutors, and judges, we suggest providing a supplement to the police record for lineups. The aspired supplement should clarify that the post-decision confidence judgment is but *one* piece of information when assessing a lineup decision. It is important to note that considering the post-decision confidence judgement does not alter the role of established impact factors of lineup performance (see e.g., [28,54,55] for reviews of these factors). If there are no serious limitations to the reliability of the lineup (i.e., the lineup occurred under pristine conditions), a highly confident lineup decision indicates a higher diagnostic value than a less confident lineup decision. Or, in other words, the likelihood that a positive lineup decision is correct is higher when the witness’ post-decision confidence judgment is high, compared with lower.

Lawyers may object that only identification decisions made with 100% post-decision confidence would meet the threshold of a court for relying on an identification decision and that lineup decisions made with lower post-decision confidence than 100% should be discarded. We recommend a different approach. If calibration is perfect, 90% of the witnesses who provide a 90% post-decision confidence rating should be accurate, 80% of those who make an 80% judgment and so on. The posterior-by-prior Bayesian curves we conducted here are another way of illustrating differences in the diagnosticity of high vs. low post-decision confidence positive identifications (see Fig 2). In our experiment, high post-decision confidence selections were highly accurate (95%) even at a 10% base rate of the suspect actually being the perpetrator. At this base rate, low post-decision confidence identifications were also diagnostic of guilt but were more error prone (72% accuracy; see [10,12] for similar findings). Both calibration and the posterior-by-prior Bayesian curves showed that the post-decision confidence judgment can provide information on how to *weigh* the identification decision. Investigators and judges can combine this information with the diagnosticity of other evidence in a given case. Additionally, the post-decision confidence judgment can help assigning diagnostic value to the lineup decisions of different witnesses–as long as those witnesses viewed the crime and the lineup under similar conditions.

Practitioners frequently express concern that group probabilities do not inform them about an individual, because individuals are unique. Approaching this issue from a medical treatment perspective can help understand why group probabilities matter in individual cases (cf. [56]). Consider a patient who has recently experienced severe pulmonary embolism. Imagine there are two treatment options with similar side effects. Controlled studies found an 85% survival rate for treatment A and a 60% survival rate for treatment B. The logic that group probabilities would be irrelevant to this patient would incorrectly suggest that the patient has no legitimate grounds for selecting the former treatment over the latter. There are no guarantees that the patient will survive if he chooses for treatment A, but his chances are better than when he chooses treatment B. A similar logic can be applied in the legal context. There are no guarantees that a highly confident witness has made a correct identification decision. But the probability that she has made a correct identification decision is higher than if she made a low confidence decision–pristine lineup conditions given.

## Supporting information

S1 Fig (TIF)

S1 File (DOCX)
