# Supplementary figures and images for: The confidence-accuracy relationship for lineup decisions holds for the Dutch identification procedure
Source: PLoS One. 2023 Apr 11;18(4):e0284205. doi: 10.1371/journal.pone.0284205 (PMC10089327; doi:10.1371/journal.pone.0284205)

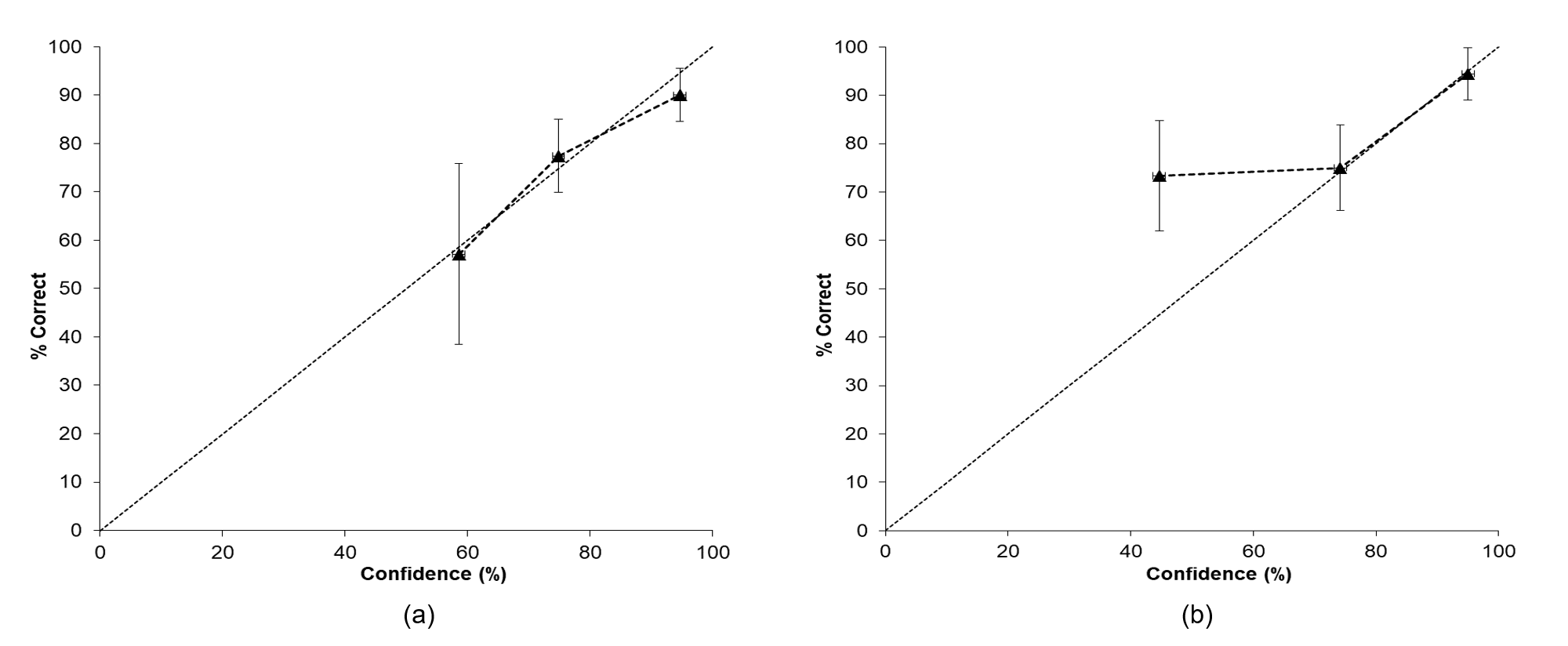

Supplement: S1 Fig — (TIF) [file pone.0284205.s001.tif]
