# Supplementary material for: The confidence-accuracy relationship for lineup decisions holds for the Dutch identification procedure
Source: PLoS One. 2023 Apr 11;18(4):e0284205. doi: 10.1371/journal.pone.0284205 (PMC10089327; doi:10.1371/journal.pone.0284205)
Supplement: S1 File — (DOCX) [file pone.0284205.s002.docx]

### Witnesses’ Confidence-Accuracy-Characteristic Analyses. In the next step, we created the confidence-accuracy-characteristic (CAC) curve for positive identifications. Compared to choosers’ calibration curve, this graph considers suspect selections, but not foil selections. Fig S1 (a) shows that all three confidence categories follow the diagonal line neatly, displaying excellent calibration in all three confidence categories with slight overconfidence in the highest confidence category.

**Fig S1. Choosers’ Confidence-Accuracy-Characteristics Curves (and Standard Error) for Eyewitness Ratings (a) and Confidence-Accuracy-Characteristics Curves (and Standard Error) for Lineup Administrator’s ratings (b)**

### Confidence-Accuracy-Characteristic Analyses for Lineup Administrators’ Confidence Ratings. We also created the CAC curve for administrators’ confidence ratings for positive identifications. Fig S1(b) shows that this choosers’ curve differs from the witness’ curve only in the lowest category, where lineup administrators display high levels of underconfidence, while perfect and almost calibration is displayed in the mid and high confidence categories, respectively.
